# Association of *VPREB1* Gene Copy Number Variation and Rheumatoid Arthritis Susceptibility

**DOI:** 10.1155/2020/7189626

**Published:** 2020-10-07

**Authors:** Muhammad Muaaz Aslam, Peter John, Kang-Hsien Fan, Attya Bhatti, Eleanor Feingold, F. Yesim Demirci, M. Ilyas Kamboh

**Affiliations:** ^1^Atta-ur-Rahman School of Applied Biosciences, National University of Sciences and Technology, Islamabad, Pakistan; ^2^Department of Human Genetics, Graduate School of Public Health, University of Pittsburgh, Pittsburgh, PA, USA

## Abstract

**Objective:**

Copy number variation (CNV) is a structural variation in the human genome that has been associated with multiple clinical phenotypes. B cells are important components of rheumatoid arthritis- (RA-) mediated immune response; hence, CNV in the regulators of B cells (such as VPREB1) can influence RA susceptibility. In this study, we aimed to explore the association of CNV in the *VPREB1* gene with RA susceptibility in the Pakistani population.

**Methods:**

A total of 1,106 subjects (616 RA cases, 490 healthy controls) were selected from three rheumatology centers in Pakistan. *VPREB1* CNV was determined using the TaqMan® CN assay (Hs02879734_cn, Applied Biosystems, Foster City, CA, USA), and CNV was estimated by using CopyCaller® (version 2.1; Applied Biosystems, USA) software. Odds ratio (OR) was calculated by logistic regression with sex and age as covariates in *R*.

**Results:**

A significant association between >2 *VPREB1* CNV and RA risk was observed with an OR of 3.92 (95% CI: 1.27 - 12.12; *p* = 0.01746) in the total sample. Whereas <2 CNV showed a significantly protective effect against RA risk in women with an OR of 0.48 (95% CI: 0.29-0.79; *p* = 0.00381).

**Conclusion:**

CNV > 2 of *VPREB1* is a risk factor for RA in the total Pakistani population, while CNV < 2 is protective in women.

## 1. Introduction

Rheumatoid arthritis (RA) is a systemic autoimmune disease. Production of autoantibodies (anticyclic citrullinated peptide and rheumatoid factor) and inflammation of joints and synovium are the hallmarks of RA [1]. RA leads to the onerous socioeconomic burden, continuous fatigue, restricted mobility due to joint damage, disability, and, in worst cases, amputation and early death [2]. RA prevalence varies across the globe in different populations, but on average, it affects 1% of the general population and occurs more frequently in women than men [3]. The interplay of multiple genetic and environmental factors leads to the onset of RA. The genetic component accounts for 50% of the RA risk [4].

Copy number variations (CNVs) are the structural variations in the human genome, defined as the stretches of DNA sequences (1 kb or longer) that are present in varied copy numbers (CN) as compared to a reference genome [5]. CNV can be a deletion or duplication of homologous sequences at multiple locations in a genome [6]. CNVs are understudied complex variations, and just like single-nucleotide polymorphisms (SNPs), their prevalence in the general population is more than 1% [7]. Gene CNV can alter the gene expression that can lead to phenotype aberrations. Recent data have suggested a significant association of gene CNV with multiple complex diseases [8], including RA [9].

B cells are antigen-presenting cells and an integral part of humoral adaptive immunity in RA-mediated immune response [10]. B cells infiltrate the synovium and approximately 60% of synovium samples harvested from RA patients have revealed the presence of B cells [11]. B cell receptor (BCR) is the immunoglobulin receptor present on the surface of B cells. The immature form of BCR is known as the “precursor of B cell receptor” (pre-BCR). Pre-BCR and BCR together regulate the B cell activation, homeostasis, function, and differentiation [12]. BCR is made up of immunoglobulin (Ig) heavy chain (HC) and light chain (LC) and its precursor form (pre-BCR) is composed of two Ig-HC and two surrogate-LC (SLC). The SLC of pre-BCR is composed of VpreB (also known as VPREB1) and *λ*5 polypeptides [13, 14]. Since SLC helps in maintaining the tolerance to self-antigens, its expression has been reported in the autoreactive B cells that were accumulated in arthritic joints [15].

In humans, VPREB1 protein is encoded by the *VPREB1* gene located on the short arm (q) of chromosome 22 (22q11). This region has been reported to be associated with multiple diseases, such as juvenile rheumatoid arthritis-like polyarthritis, chronic autoimmune arthritis, and RA [16–18]. To our knowledge, only one study has reported the association of *VPREB1* CNV with RA in Koreans [16]. In order to better understand the role of CNV in RA in this region, we have examined the association of *VPREB1* CNV with RA risk in the Pakistani population.

## 2. Materials and Methods

### 2.1. Study Subjects

A total of 1,106 subjects (616 RA cases, 490 healthy controls) were recruited for this study. Blood samples of RA cases (mean age ± SD = 43.68 ± 12.15, 77.9% women) were collected from the Pakistan Institute of Medical Sciences (*n* = 230), military hospital (*n* = 298), and Rehmat Noor Clinic (*n* = 88) in Islamabad, Pakistan. RA cases were diagnosed by a rheumatologist and met the American College of Rheumatology (ACR) 1987 criteria for the classification of RA [19]. Control subjects (mean age ± SD = 38.3 ± 12.26, 45% women) were recruited from the same region as the RA patients, and they were free from any autoimmune disease at the time of enrollment. Both cases and controls were recruited during the same period from September 2015 to May 2017. Blood samples were collected in EDTA-coated tubes to prevent coagulation and processed shortly after the collection. This study was approved by the institutional review board (IRB) of the University of Pittsburgh, Pittsburgh, PA, USA (IRB no. PRO12110472).

### 2.2. Genomic DNA Extraction

A standard phenol-chloroform extraction method or GeneJET Whole Blood Genomic DNA Purification (Thermo Scientific USA) was used to extract genomic DNA from the whole blood and quantified using the NanoDrop™ 2000 spectrophotometer (Thermo Scientific USA).

### 2.3. Measurement of *VPREB1* Copy Number

**Figure 1:**
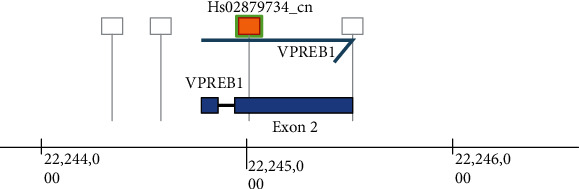
Genomic map of the TaqMan® assay. Image downloaded from https://www.thermofisher.com/us/en/home.html.

It was evident from the primer sequences published in an earlier study [16] that exon 2 of *VPREB1* is a potential target site of CNV. In the current study, *VPREB1* CNVs were determined using TaqMan® (Applied Biosystems, Foster City, CA, USA) CNV assay where the hydrolysis probe is also located within exon 2 of *VPREB1* (Chr.22:22244780-22245515, Build GRCh38), which amplified a fragment size of 72 base pairs (Figure 1). 384-well-dried DNA plates were used to run quantitative polymerase chain reaction (qPCR) using FAM-MGB dual-labeled hydrolysis probe (Hs02879734_cn) with RNaseP (4403326, VIC-TAMRA dual-labeled probe) as a reference assay in a duplex reaction, following the manufacturer's guidelines. Three reference samples were added to each 384-well plate for the validation of constant reaction conditions and CNV assignment. All samples were tested in quadruplicate. QuantStudio™ 12K Flex system (Applied Biosystems, Thermo Fisher Scientific) was used to run qPCR, and fluorescence signals were normalized to ROX reference dye. qPCR data were analyzed using a 0.2-cycle threshold and Ct autobaseline as recommended by the manufacturer. qPCR data was exported and CNVs were assigned using CopyCaller® (version 2.1; Applied Biosystems, USA) software. CopyCaller® software provides calculated and predicted (discrete) CNV assignment using qPCR data and offers additional quality control (QC) checkpoints, like confidence estimate and *Z*-score against each CNV call. All plates were analyzed separately to minimize the effect of experimental variations on different plates. *Z* − score < 2.66, zero-copy *Δ*Ct threshold, copy number range (CNR=difference between minimum and maximum copy number detected among replicate samples) less than 0.5 (CNR < 0.5), and exclusion of samples that have Ct greater than 32.0 for reference assay (VIC-labeled RNaseP) were used as QC measures during the analysis.

### 2.4. Statistical Analysis

Logistic regression was applied to test the association between RA susceptibility and CNV. Subjects with 2 CNV of *VPREB1* were treated as the reference group, which was compared to subjects with CNV < 2 or CNV > 2 groups. Age and sex were used as the covariates in the regression model. Odds ratios (ORs), 95% confidence intervals (CI), and the corresponding *p* values were calculated. Association of *VPREB1* CNVs with rheumatoid factor (RF) and anticyclic citrullinated peptide (anti-CCP) seropositivity was also measured by logistic regression using sex and age as covariates. All the statistical analyses were performed using R version 3.6.14 (https://www.r-project.org).

## 3. Results

**Figure 2:**
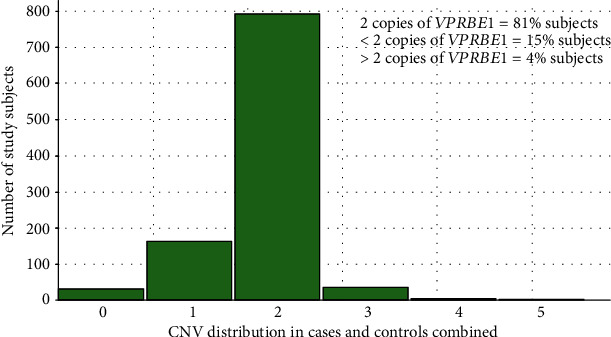
CNV distribution in cases and controls combined.

**Figure 3:**
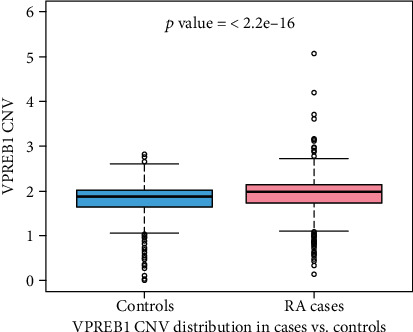
CNV distribution in cases and controls. The *p* value was calculated from a two-sample *t*-test.

The demographic information of the study subjects is presented in Table 1. For each sample, the predicted CNV was categorized into three groups: less than two (CNV < 2), equal to two (CNV = 2), or greater than two (CNV > 2). The three reference samples did not show any variation across all the tested plates, indicating no bias in the CNV assignment. The distribution of CNV in cases and controls combined is illustrated in Figure 2. About 81% (896/1106) of the combined sample showed two copies of *VPREB1*, 15% (166/1106) had <2 copies, and 4% (44/1106) had >2 copies. Only <1.0% (*n* = 6/616) of RA cases and 1.2% (*n* = 6/490) of controls showed the complete deletion (0 copies) of the *VPREB1* allele. The highest CNV detected in controls was 3 (1.2%) as compared to RA cases that showed 4 (0.5%) and 5 (0.1%) copies (Figure 3).

The distribution of three CNV groups (<2, 2, and >2) between cases and controls is shown in Table 2. The prevalence of >2 copies was significantly higher in RA cases (5.6%; 35/616) as compared to controls (1.2%; 6/490) with an OR of 3.92 (95% CI: 1.27 to 12.12; *p* = 0.0175). On the other hand, the incidence of <2 copies was lower in cases than controls: 13.2% (81/616) vs. 16.8% (82/490); *p* = 0.056). To assess the gender-specific effect of *VPREB1* CNV on RA susceptibility, the data were analyzed separately in men and women by logistic regression using age as a covariate. A significant association was observed only in women with CNV < 2 copies having an OR of 0.48 (95% CI: 0.29 to 0.79; *p* = 0.00381). Although the same risk pattern was observed in both men and women as in the total sample, it was not statistically significant, most probably because of the small sample sizes in subgroups (Table 2). Logistic regression analysis of *VPREB1* CNV with anti-CCP and RF seropositivity did not show any significant association either in the total sample (Table 3) or sample stratified by gender (data not shown).

## 4. Discussion

B cells are the key mediators of the innate and adaptive immune system. Any alternation in B cells can initiate a range of disorders [20]. The mechanism behind the production of autoreactive B cells in RA patients and the factors that elicit this process is still not clear. The role of pre-BCR in the regulation of B cell production and the positive and negative selection of autoreactivity make it an important component of autoimmunity [21]. As *VPREB1* is involved in the development of pre-BCR, any impairment in its expression can ultimately lead to B cell immunodeficiency [22].

Structural variations, such as CNVs, are the major source of genetic diversity in humans. CNVs can influence the expression of a gene, and they can contribute to pathological conditions in dosage-sensitive genes [23]. CNVs in different genomic regions have been associated with various diseases, including RA. CNVs affecting *CCL3L1* [24], *FCGR3B* [25, 26], *C4B* [27], and *LCE3B* [28] genes have been reported to influence RA susceptibility. *VPREB1* is located on chromosome 22q11 and CNV in this gene has been reported to be associated with RA in one Korean study that included 473 cases and controls [16].

In this study, we examined a much larger sample (*n* = 1,106) from a Pakistani population to investigate the association of *VPREB1* CNV with RA susceptibility. Our results are opposite to the previously published study in Koreans where <2 CNV was associated with an increased RA risk and > 2 CNV was protective [16]. In our sample, while >2 CNV was significantly associated with RA risk having an OR of 3.92 (95% CI: 1.27-12.12; *p* = 0.017), and <2 CNV appears to be protective against RA, especially in women where it achieved statistical significance (OR = 0.48; 95% CI: 0.29–0.79; *p* = 0.0038). Various factors may explain the difference in results between the two studies, including differences in methodology (e.g., probe choice for qPCR), ethnic origin and/or clinical features of the study population, sample size, frequency, and/or effect size of CNVs. The target region in both studies appears to be the exon 2 of *VPREB1.* However, the overall CNV distribution pattern was significantly different between the Pakistani and Korean samples. While the prevalence of CNV < 2 was higher in Pakistanis than in Koreans (15% vs. 7%), the prevalence of CNV > 2 was lower in Pakistanis than in Koreans (4% vs. 10%). Nevertheless, the less common CNV in both samples was associated with RA risk (>2 in Pakistanis, but <2 in Koreans). This opposite association in two different ethnic groups suggests that CNV in this region is perhaps not causative but rather in linkage disequilibrium (LD) with functional variant(s), which have different LD patterns in these groups. More follow-ups of such association studies in larger samples along with targeted sequencing in this region may provide a definitive answer. Moreover, the interpretation of *VPREB1* CNV analysis is complicated by the fact that this gene is located within the immunoglobulin *λ*-light chain region that undergoes complex somatic rearrangements, specifically in B cells during their development. Significant heterogeneity in B cell count/population has been reported in RA depending on the clinical subtype, disease activity, disease duration, and type of therapy received [29, 30]. Unfortunately, no information related to the B cell count/population was available in our sample to allow a comparison with the previous study. Clearly, additional studies on large samples from different population groups are needed to address the role of *VPREB1* CNV in RA susceptibility.

## 5. Conclusions

In summary, we examined the association of *VPREB1* CNV and RA susceptibility in Pakistanis and found a significant association of >2 *VPREB1* CNV with an increased RA risk in the total sample and a protective effect in women. Our data also highlight a potential ethnic-specific association of *VPREB1* CNV in exon 2 that needs to be confirmed in larger and independent samples from different population groups.

## Figures and Tables

**Table 1:** Demographic information of study subjects.

	RA cases (*N* = 616)	Healthy controls (*N* = 490)
Mean age, year ± SD	43.68 ± 12.15	38.3 ± 12.26
Women (%)	77.9%	45%

**Table 2:** Association results of *VPREB1* CNV and RA susceptibility.

*VPREB1* CNV	RA cases (*n* = 616)	Controls (*n* = 490)	All subjects		Men		Women	
			OR^∗^ (95% CI)	*p* value	OR^∗∗^ (95% CI)	*p* value	OR^∗∗^ (95% CI)	*p* value
<2	13.2%	16.8%	0.66 (0.43 to 1.01)	0.05622	1.25 (0.61 to 2.55)	0.538	0.48 (0.29 to 0.79)	0.00381
2	81.2%	82%	1		1		1	
>2	5.6%	1.2%	3.92 (1.27 to 12.12)	0.01746	2.67 (0.58 to 12.34)	0.208	6.75 (0.9 to 50.69)	0.0635

OR^∗^ was adjusted for age and sex. OR^∗∗^ was adjusted for age only.

**Table 3:** Association results of *VPREB1* CNV with anti-CCP seropositivity.

*VPREB1* CNV	Anti-CCP		RF factor	
	OR^∗^ (95% CI)	*p* value	OR^∗^ (95% CI)	*p* value
<2	1.51 (0.71 to 3.2)	0.29	1.37 (0.65 to 2.92)	0.41
2	1		1	
>2	1.27 (0.42 to 3.82)	0.67	0.68 (0.26 to 1.74)	0.42

OR^∗^ was adjusted for age and sex.

## Data Availability

The data used to support the findings of this study are included in the article.
